# Predictive value of integrated ^18^F-FDG PET/MRI in the early response to nivolumab in patients with previously treated non-small cell lung cancer

**DOI:** 10.1136/jitc-2019-000349

**Published:** 2020-04-28

**Authors:** Yukihiro Umeda, Miwa Morikawa, Masaki Anzai, Shingo Ameshima, Maiko Kadowaki, Yuko Waseda, Hiroko Shigemi, Tetsuya Tsujikawa, Yasushi Kiyono, Hidehiko Okazawa, Tamotsu Ishizuka

**Affiliations:** 1 Third Department of Internal Medicine, Faculty of Medical Sciences, University of Fukui, Fukui, Japan; 2 Department of Internal Medicine, Sakai Municipal Mikuni Hospital, Fukui, Japan; 3 Biomedical Imaging Research Center, University of Fukui, Fukui, Japan

**Keywords:** MRI, nuclear medicine, oncology, PET

## Abstract

**Background:**

The early response to treatment with immune-checkpoint inhibitors is difficult to evaluate. We determined whether changes in integrated [^18^F]-fluoro-2-deoxy-D-glucose positron emission tomography/MRI (^18^F-FDG PET/MRI) parameters after the first 2 weeks of antiprogrammed death-1 antibody nivolumab therapy could predict the response of patients with non-small cell lung cancer (NSCLC).

**Methods:**

Twenty-five patients with previously treated NSCLC were enrolled prospectively and underwent ^18^F-FDG PET/MRI before and at 2 weeks after nivolumab therapy. Changes in maximal standardized uptake value, total lesion glycolysis (ΔTLG) and apparent diffusion coefficient (ΔADC) between the two scans were calculated and evaluated for their associations with the clinical response to therapy.

**Results:**

The disease control rate was 64%. Patients with non-progressive disease (non-PD) had significantly decreased TLG, increased ADC_mean_ (ie, negative ΔADC_mean_) and lower ΔTLG+ΔADC_mean_ than patients with PD. Among the parameters tested, receiver operating characteristic curve analysis revealed that a cut-off value of 16.5 for ΔTLG+ΔADC_mean_ had the highest accuracy (92%) for distinguishing between patients with non-PD and PD. A ΔTLG+ΔADC_mean_ value <16.5 was significantly associated with longer median progression-free survival (9.0 vs 1.8 months, p<0.00001) and overall survival (23.6 vs 4.7 months, p=0.0001) compared with ΔTLG+ΔADC_mean_ value ≥16.5. A multivariate Cox model revealed that ≥16.5 ΔTLG+ΔADC_mean_ was an independent predictor of shorter progression-free survival (HR 37.7) and overall survival (HR 9.29).

**Conclusions:**

A combination of ΔTLG and ΔADC_mean_ measured by integrated ^18^F-FDG PET/MRI may have value as a predictor of the response and survival of patients with NSCLC following nivolumab therapy.

**Trial registration number:**

UMIN 000020707.

## Background

Programmed death-1 (PD-1) is one of several proteins that serve as checkpoints for immune cell activation. Primed T cells expressing PD-1 are inactivated by engagement of its ligand programmed death ligand 1 (PD-L1) expressed on tumor cells, thereby promoting tumor growth and metastasis.^1^ The anti-PD-1 antibody nivolumab was recently approved for previously treated non-small cell lung cancer (NSCLC). Several phase III studies have shown that nivolumab confers a superior survival benefit compared with docetaxel, a standard second-line cytotoxic agent, in both patients with squamous and non-squamous NSCLC.^2 3^ Based on these results, nivolumab is recommended as a standard second-line regimen in the recent guidelines for NSCLC.^4^

The proportion of tumor cells expressing PD-L1, as assessed by immunohistochemistry, is considered a predictive biomarker for the efficacy of anti-PD-1 therapy. However, objective response rates of only 19% and 37% have been reported for patients with high PD-L1-expressing (≥10% cells) squamous and non-squamous NSCLC, respectively, following nivolumab treatment.^2 3^ Several case reports have described a phenomenon known as pseudoprogression, in which initial tumor progression is followed by shrinkage during continuous treatment with immune-checkpoint inhibitors^5 6^; notably, this is not seen with cytotoxic drugs or other targeted agents. For these reasons, it is currently difficult to accurately predict or assess the early response to immune-checkpoint inhibitors. Additionally, these inhibitors are associated with debilitating side effects and are very expensive (eg, nivolumab is currently US$15,329 per month). Therefore, there is an urgent need to identify new predictive biomarkers of the early response to immune-checkpoint inhibitors, which will help to curtail ineffective and potentially toxic therapy and reduce unnecessary costs.

Currently, [^18^F]-fluoro-2-deoxy-d-glucose (^18^F-FDG) positron emission tomography (PET) is widely used for the diagnosis of malignancy, and it has recently been used to assess the early response of patients with NSCLC to chemotherapy,^7 8^ targeted agents^9^ and chemoradiotherapy.^10 11^ Diffusion-weighted MRI has also been used to assess the early response to chemotherapy in patients with lung cancer.^7^ The apparent diffusion coefficient (ADC) is a commonly used functional parameter and surrogate marker of tumor cellularity and proliferation in MRI,^12 13^ and a change in ADC has been reported to precede a reduction in tumor size.^14^ Furthermore, integrated PET/MRI technology has recently become available and can provide useful information about metabolic activity and tumor cellularity simultaneously. In the present study, we sought to determine whether changes in integrated ^18^F-FDG PET/MRI parameters after the first 2 weeks of nivolumab therapy could accurately discriminate between responders and non-responders, as assessed by Response Evaluation Criteria in Solid Tumor (RECIST) criteria, and/or predict patient survival.

## Materials and methods

### Study design and patient selection

The clinical trial was registered at www.umin.ac.jp/ctr/. We prospectively enrolled 25 consecutive patients treated at the University of Fukui Hospital from January 2016 to February 2018, and the protocol-defined final analysis was performed on August 31, 2018. Eligibility criteria included histologically/cytologically confirmed advanced or recurrent NSCLC measurable by RECIST, one or more prior systemic chemotherapy regimens and an Eastern Cooperative Oncology Group performance status (ECOG-PS) of 0 to 2. Exclusion criteria included a fasting serum glucose level >150 mg/dL, a metallic device in the body, claustrophobia, obvious interstitial pneumonia or pulmonary fibrosis on chest X-ray, collagen vascular disease or autoimmune disease.

All patients were administered nivolumab intravenously at a dose of 3 mg/kg body weight every 2 weeks until disease progression or discontinuation of treatment because of adverse events. ^18^F-FDG PET/MRI and CT were performed within 2 weeks before (baseline) and between 11 and 14 days (2 weeks) after initiation of nivolumab therapy. When disease control was evident, a PET/MRI scan was undertaken between 51 and 61 days (8 weeks) after initiation of nivolumab therapy. Tumor response was assessed using RECIST V.1.1^15^ at week 2 and at least every 6 weeks thereafter.

### CT and image analysis

CT of the chest and abdomen was performed using a whole-body CT scanner with a multidetector row CT system (SOMATOM Force, Siemens Healthcare, Erlangen, Germany or Discovery CT750 HD, GE Healthcare, Waukesha, Wisconsin, USA). Contiguous 3.0 mm thick sections were obtained at 3.0 mm intervals from the apices to pelvis. CT-based responses were calculated from the sum of the diameters according to RECIST V.1.1.^15^

### Integrated PET/MRI

^18^F-FDG PET/MRI was performed on an integrated 3-Tesla PET/MR scanner (SIGNA PET/MR, GE Healthcare). Patients fasted for at least 6 hours prior to receiving an intravenous injection of 185 MBq of ^18^F-FDG, which is a commonly used dose for clinical ^18^F-FDG PET scanning in Japan. Fifty minutes after the injection, patients were transferred to the PET/MR scanner. PET acquisition was performed in three-dimensional (3D) mode with 10 min/bed position (89 slices/bed) in two beds with a 44-slice overlap to cover the thorax and upper abdomen. A 2-point Dixon 3D volumetric interpolated T1-weighted fast spoiled gradient echo sequence (repetition time (TR)/echo time (TE)1/TE2, 4.0/1.1/2.2 ms; FOV, 50×37.5 cm; matrix, 256×128; slice thickness/overlap, 5.2/2.6 mm; 120 image/slab imaging time, 18 s) was acquired at each table position and used to generate MR attenuation correction maps. Dixon-based MR attenuation correction recognizes body tissues as soft tissue, fat and air. PET data were reconstructed with ordered subset expectation maximization selecting 32 subsets and 3 iterations, and postsmoothing with a 4 mm Gaussian filter. Reconstructed images were then converted to semiquantitative images corrected for the injected dose and the subject’s body weight (standardized uptake value (SUV)).

For thoracic MRI, T2-weighted images were acquired in the transaxial plane. The following T2-weighted image parameters were used: TR/TE, 7800/182.3 ms; slice thickness, 6 mm; slice spacing, 0 mm; flip angle, 120°; field of view (FOV), 460 mm×460 mm; matrix, 384×384; number of excitations (NEX), 1.5 and bandwidth, 55.56 kHz. Finally, respiratory-gated diffusion-weighted images were obtained in the transaxial plane with b-values of 0 and 800 s/mm^2^. The scan parameters were as follows: effective TR/TE, 12,000/70.2 ms; slice thickness, 5 mm; slice spacing, 0.5 mm; flip angle, 90°; FOV, 384 mm×384 mm (phase FOV=0.8); matrix, 128×128; NEX, 8; bandwidth, 250 kHz and acquisition time, 5 min 12 s.

### PET image analysis

The ^18^F-FDG PET images were interpreted independently by one experienced radiologist and one oncologist who were blinded to the clinical data. Using an automatic registration software package (Advantage Workstation; GE Healthcare), volumes of interest (VOIs) were placed on the primary and metastatic tumor for the semiquantitative analysis. The VOIs on pretreatment PET images were automatically registered and used in the repeated study at 2 and 8 weeks after treatment. The tumor contour was delineated to include voxels presenting SUV values >40% of maximal SUV (SUV_max_). The extracted tumor volume was defined as the metabolic tumor volume, and total lesion glycolysis (TLG) was calculated as the metabolic tumor volume multiplied by the average SUV (SUV_mean_) within the extracted tumor area. For each patient, the lesion with the highest SUV_max_ on the pretreatment PET images was selected as the representative lesion.

### MRI analysis

The ADC map was derived from the diffusion-weighted images as follows:

ADC (mm^2^/s)=−ln[S(*b*)/S(0)]/*b,* where *b* is the diffusion weighting factor and S(*b*) and S(0) are signal intensities with and without diffusion-sensitizing gradients, respectively. In this study, *b*=800 was applied. For each patient, the lesion with the maximum diameter was selected as the representative lesion. Circular regions of interest were drawn within the lesion and the average ADC (ADC_mean_) was calculated.

### Response assessment

CT-based responses were calculated from the sum of the diameters according to RECIST V.1.1.^15^ To distinguish the immune-related response (ie, pseudoprogression) from tumor progression, the definition of confirmation of progressive disease (PD) represented an increase in the sum of diameters ≥20% compared with the nadir at two consecutive points at least 4 weeks apart in the absence of rapid clinical deterioration.

Percentage changes (Δ) in the sum of the diameters, SUV_max_ and TLG from pretreatment (scan 1) to 2 weeks after treatment initiation (scan 2) were calculated as follows:

Δparameter (%)=(scan 2− scan 1)×100/scan 1.

Unlike other parameters, the ADC_mean_ increased between scans in responders; therefore, the percentage change in ADC_mean_ from scan 1 to scan 2 was calculated as follows:

ΔADC_mean_ (%) = (scan 1−scan 2)×100/scan 1.

The sum of the changes in TLG (ΔTLG) and ADC_mean_ (ΔADC_mean_) is presented as ΔTLG+ΔADC_mean_.

^18^F-FDG PET-based responses were evaluated according to the Positron Emission Tomography Response Criteria in Solid Tumors V.1.0^16^ and European Organisation for the Research and Treatment of Cancer (EORTC) 1999 criteria.^17^ SUV normalized by lean body mass (SUL) peak was measured using syngo.via (Siemens Healthcare).

PET-based progressive metabolic disease (PMD) at 8 weeks was defined as an increase in SUV_max_ of >25% within the tumor region defined in the baseline scan or the appearance of new ^18^F-FDG uptake in metastatic lesions according to the EORTC 1999 criteria.^17^ The patients were dichotomized into those with PMD and others (stable metabolic disease, partial metabolic response and complete metabolic response) to evaluate progression-free survival (PFS) using Kaplan-Meier methods. Patients with confirmed PD before or within 2 weeks after the 8-week ^18^F-FDG PET scan were excluded from this analysis.

### PD-L1 expression analysis

Tumor expression of PD-L1 was measured using immunohistochemistry (IHC) at LSI Medience (Tokyo, Japan) using the PD-L1 IHC 22C3 pharmDx assay (Agilent, Santa Clara, California, USA) and a Dako Autostainer Link 48 platform (Dako, Carpenteria, California, USA).

For evaluation of PD-L1 staining, the tumor proportion score (TPS) was calculated as the percentage of at least 100 viable tumor cells with complete or partial membrane staining. PD-L1 expression was interpreted by one trained pathologist and two trained histotechnologists employed by the commercial vendor (LSI Medience).

### Statistical analysis

The primary end point was the predictive value of serial ^18^F-FDG PET/MRI findings, including SUV_max_, TLG and ADC_mean_, for tumor response to nivolumab therapy. The secondary end point was the predictive value of those parameters for PFS, defined as the time from treatment initiation to disease progression or death, and overall survival (OS), defined as the time from treatment initiation to death from any cause.

Categorical data were compared using Fisher’s exact test or a χ^2^ test as appropriate. The Mann-Whitney U test was used for comparisons between groups. Receiver operating characteristic (ROC) curves were constructed, and the corresponding areas under the curve were calculated to determine the most discriminative serial ^18^F-FDG PET/MRI parameter and their optimal thresholds. The diagnostic accuracy of the parameters was compared using McNemar’s test. OS and PFS were evaluated using the Kaplan-Meier method with a log-rank test. All analyzes were performed using SPSS Statistics V.22.0 (IBM, Armonk, New York, USA). P values <0.05 were considered to be statistically significant.

## Results

### Patient characteristics

A total of 25 patients (median age 70, range 46–84 years) with previously treated NSCLC underwent integrated ^18^F-FDG PET/MRI before and after nivolumab therapy. The average interval (days±SD) between ^18^F-FDG PET/MRI at baseline and at weeks 2, 8 and initiation of nivolumab therapy was −1.56±2.81, 12.84±0.94 and 56.81±4.46 days, respectively. The average interval between the CT scan at baseline, week 2 and initiation of nivolumab therapy were −3.80±4.48 and 12.68±1.03 days, respectively. Any adverse events by the PET/MRI was not observed. Table 1 summarizes the patient characteristics. Of the 25 patients, 12 had adenocarcinoma, 12 had squamous cell carcinoma and 1 had NSCLC not otherwise specified. Twenty patients (80%) had tumors with quantifiable PD-L1 expression. Of these, 11 (55%) had a TPS of ≥1% and 3 (15%) had a TPS of >50%. Six patients (24%) had received three or more prior systemic treatments for advanced NSCLC.

**Table 1:** Patient characteristics (n=25)

	N (%)
Age, median (range)	70 (46–84)
Sex, male/female	18/7 (72/28)
Smoking history, yes/no	19/6 (76/24)
ECOG-PS, 0/1/2	12/12/1 (48/48/4)
Histology, ad/sq/NOS	16/8/1 (64/32/4)
Stage, III/IV/recurrence	5/17/3 (20/68/12)
Driver mutation, *EGFR*/*ALK*/none	2/1/22 (8/4/88)
Number of previous regimen, 1/2/3/≥4	11/8/4/2 (44/32/16/8)
PD-L1 expression, TPS <1%/1%–49%/≥50%/unknown	9/8/3/5 (36/32/12/20)
Confirmed response: CR/PR/SD/PD	1/8/7/9 (4/32/28/36)

ad, adenocarcinoma; ALK, anaplastic lymphoma kinase; CR, complete response; ECOG-PS, Eastern Cooperative Oncology Group performance status; EGFR, epidermal growth factor receptor; NOS, not otherwise specified; NOS, not otherwise specified; PD, progressive disease; PR, partial response; SD, stable disease; sq, squamous cell carcinoma.

### Serial CT scan and PET/MRI parameters and response assessment

Among the 25 patients, 1 (4%) achieved a complete response (CR), 8 (32%) had a partial response (PR), 7 (28%) had stable disease (SD) and 9 (36%) had PD (table 1). The objective response rate and disease control rate were 32% and 64%, respectively. No patients experienced pseudoprogression as assessed using RECIST.

**Figure 1:**
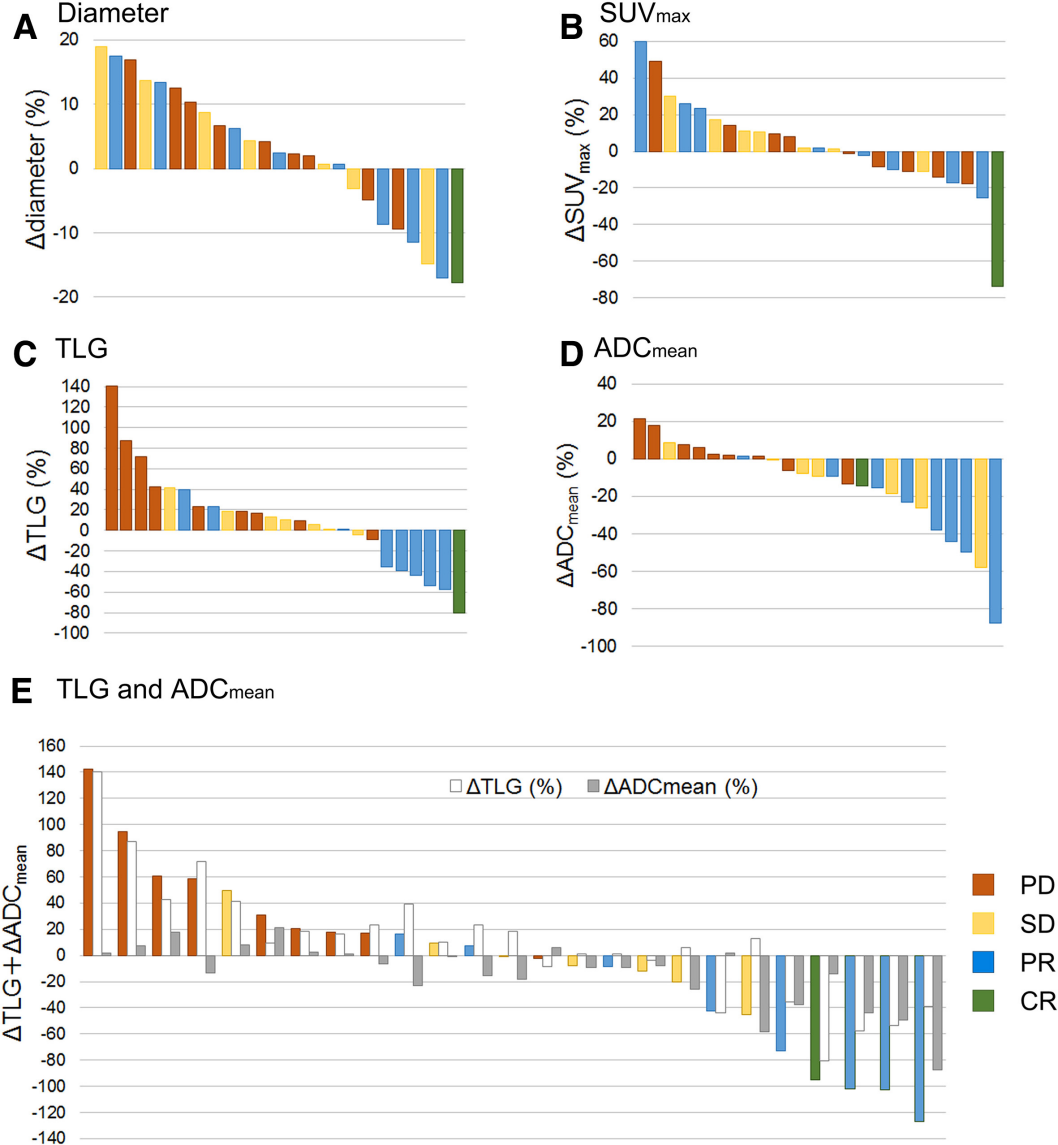
Changes from baseline in [^18^F]-fluoro-2-deoxy-D-glucose positron emission tomography/MRI parameters in nivolumab-treated patients according to treatment response. Per cent changes are shown for tumor diameter (A), maximal standardized uptake value (SUV_max_) (B), total lesion glycolysis (TLG) (C), apparent diffusion coefficient (ADC)_mean_ (D) and ΔTLG+ΔADC_mean_ (E) in all patients at 2 weeks after nivolumab therapy.

Changes in CT and PET/MRI parameters between baseline and 2 weeks after nivolumab therapy are shown in figure 1 and online supplemnetary table S1. Patients with non-PD (CR, PR and SD) had significantly decreased TLG, increased ADC_mean_ (ie, negative ΔADC_mean_) and lower ΔTLG+ΔADC_mean_ than patients with PD (figure 1C, D, E), whereas there were no significant differences between these groups in Δdiameter, ΔSUV_max_ or ΔSUL peak (figure 1A, B).

10.1136/jitc-2019-000349.supp1 Supplementary data

ROC curves were constructed to determine the appropriate cut-off values for dichotomization of patients according to Δdiameter, ΔTLG, ΔADC_mean_ and ΔTLG+ΔADC_mean_ (online supplemnetary figure S1). According to the ROC curves, 1%, 15%, −7% and 16.5 were the appropriate cut-off values for Δdiameter, ΔTLG, ΔADC_mean_ and ΔTLG+ΔADC_mean_, respectively. Online supplemnetary table S2 presents the predictive utility of these parameters for distinguishing PD and non-PD based on the ROC-determined cut-off values. Using the cut-off value of 16.5, ΔTLG+ΔADC_mean_ had the highest sensitivity, specificity and accuracy (93.8%, 88.9% and 92.0%, respectively), and its accuracy was significantly better than that of Δdiameter (92.0% vs 60.0%, p=0.027, analyzed by McNemar’s test).

10.1136/jitc-2019-000349.supp2 Supplementary data

10.1136/jitc-2019-000349.supp3 Supplementary data

## Association between changes in ^18^F-FDG PET/MRI parameters and progression-free survival

**Figure 2:**
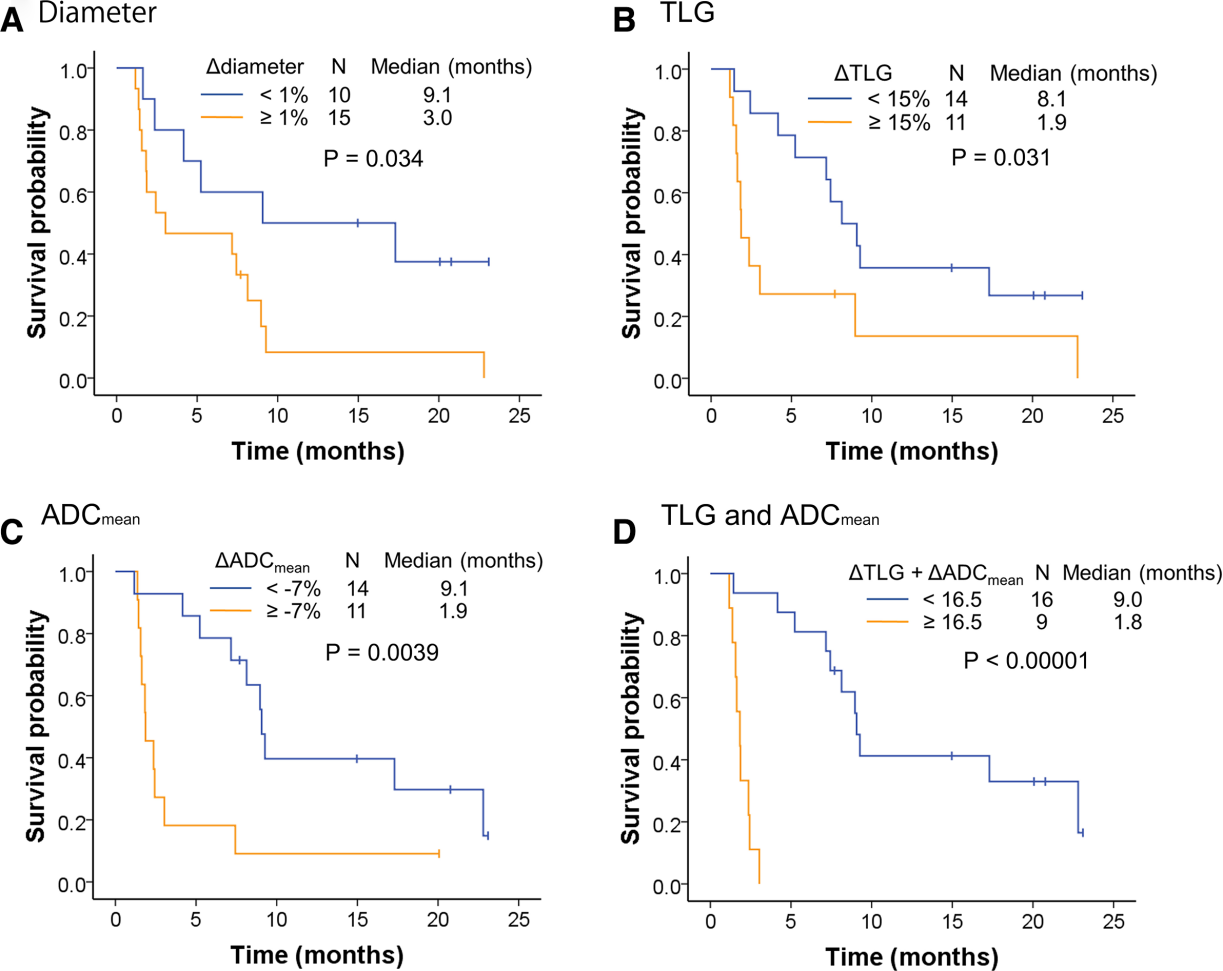
Progression-free survival of nivolumab-treated patients stratified by changes in [^18^F]-fluoro-2-deoxy-D-glucose positron emission tomography/MRI parameters. Kaplan-Meier curves of progression-free survival stratified by Δdiameter (A), total lesion glycolysis (ΔTLG) (B), apparent diffusion coefficient (ΔADC)_mean_ (C) and ΔTLG+ΔADC_mean_ (D), with cut-off values determined by receiver operating characteristic curve analysis.

The median follow-up period for all patients was 12.6 months (range 1.3–30.9 months). Twenty patients showed disease progression during the study period, and the median PFS was 7.2 months. Kaplan-Meier curves for PFS stratified by the imaging cut-off values are shown in figure 2. Significant differences in PFS were observed between the groups stratified by Δdiameter, ΔTLG and ΔADC_mean_ (figure 2A–C). The median PFS was also significantly longer for patients in the ΔTLG+ΔADC_mean_<16.5 group than those in the ≥16.5 group (9.0 vs 1.8 months, p<0.00001, figure 2D). Conversely, no significant difference in PFS was observed between patients with PD-L1 TPS of <1% or ≥1% (p=0.24, online supplemnetary figure S2a).

10.1136/jitc-2019-000349.supp4 Supplementary data

A Cox proportional hazards model was used to evaluate predictors of PFS and OS (table 2). We found that older age (≥70, HR 2.83, 95% CI 1.00 to 7.98, p=0.049) and ΔTLG+ΔADC_mean_≥16.5 (HR 37.7, 95% CI 4.50 to 316.2, p=0.00082) were indicators of shorter PFS by univariate analysis, whereas ECOG-PS, number of previous regimens, clinical stage, histology and TPS were not significant. In multivariate analysis, only higher ΔTLG+ΔADC_mean_ remained an independent predictor of PFS.

**Table 2:** Univariate and multivariate analysis of prognostic factors for progression-free (PFS) and overall survival (OS)

	PFS			OS		
	HR	95% CI	P value	HR	95% CI	P value
Univariate analysis						
Age, ≥70	2.83	1.00 to 7.98	0.049	2.58	0.79 to 8.44	0.12
ECOG-PS, 1–2	1.39	0.57 to 3.39	0.46	3.11	0.95 to 10.16	0.061
Number of previous regimens, ≥2	1.56	0.62 to 3.93	0.35	1.43	0.43 to 4.71	0.56
Stage, IV or recurrence	0.75	0.21 to 2.62	0.65	0.49	0.13 to 1.84	0.29
Histology, adenocarcinoma	1.03	0.41 to 2.60	0.95	0.48	0.16 to 1.43	0.19
TPS, <1%	1.83	0.66 to 5.09	0.25	1.89	0.56 to 6.40	0.31
ΔTLG+ΔADC_mean_, ≥16.5	37.72	4.50 to 316.2	0.00082	9.29	2.49 to 34.62	0.00091
Multivariate analysis						
ΔTLG+ΔADC_mean_, ≥16.5	37.72	4.50 to 316.2	0.00082			

ADC, apparent diffusion coefficient; ECOG-PS, Eastern Cooperative Oncology Group performance status; TLG, total lesion glycolysis; TPS, tumor proportion score.

**Figure 3:**
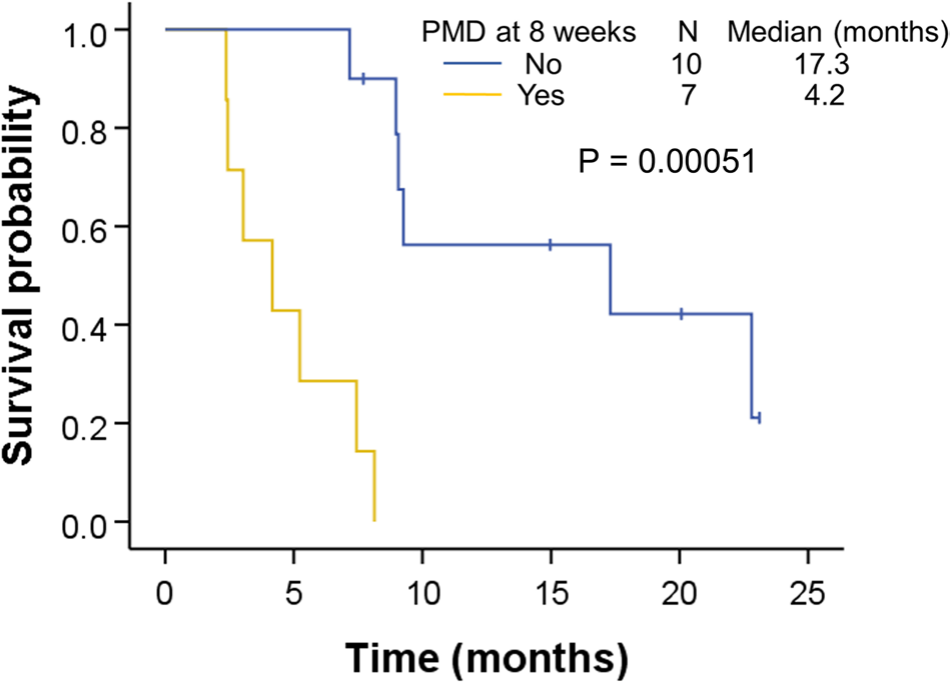
Kaplan-Meier curve of progression-free survival of nivolumab-treated patients according to [^18^F]-fluoro-2-deoxy-D-glucose positron emission tomography/MRI findings at 8 weeks. The patients were dichotomized into those with progressive metabolic disease (PMD, n=7) and others (n=10).

Among the 25 patients, 4 did not undergo ^18^F-FDG PET/MRI at 8 weeks after treatment initiation because of early disease progression. Of the remaining 21 patients, 4 showed PD in the CT images according to RECIST V.1.1 within 2 weeks after the 8-week ^18^F-FDG PET scan, which left 17 patients with no progression at 10 weeks for further analysis. Seven patients showed either increased SUV_max_ (≥25%) within the tumor region defined in the baseline scan or a new lesion on the 8-week ^18^F-FDG PET according to EORTC 1999 criteria.^17^ Kaplan-Meier PFS curves for ^18^F-FDG PET findings at 8 weeks are shown in figure 3. The median PFS for patients without relapse-associated findings was significantly longer than that of patients with PMD on ^18^F-FDG PET at 8 weeks (17.3 vs 4.2 months, p=0.000051).

### Association between changes in ^18^F-FDG PET/MRI parameters and overall survival

**Figure 4:**
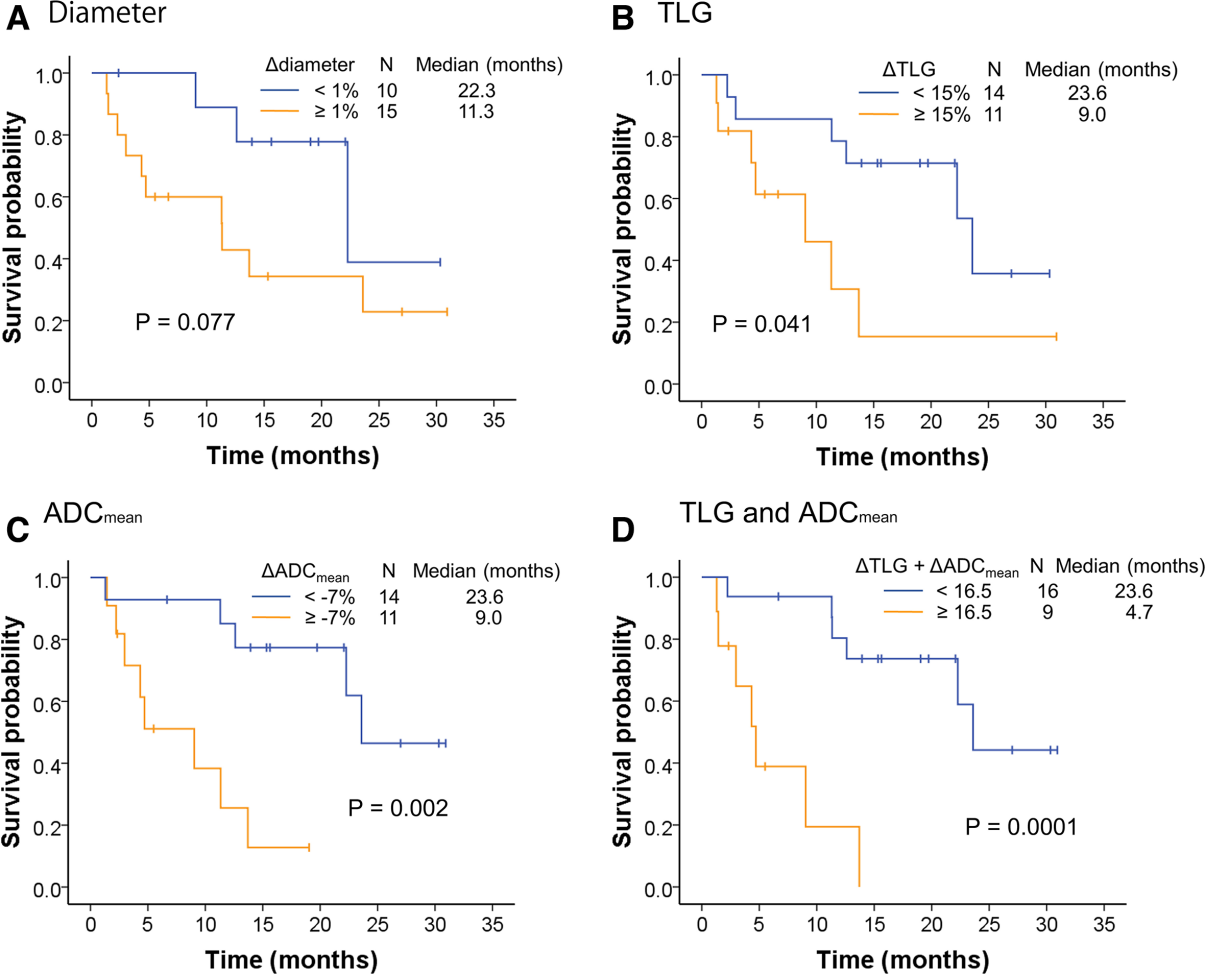
Overall survival of nivolumab-treated patients according to changes in [^18^F]-fluoro-2-deoxy-D-glucose positron emission tomography/MRI parameters. Kaplan-Meier curves of overall survival stratified by Δdiameter (A), total lesion glycolysis (ΔTLG) (B), apparent diffusion coefficient (ΔADC)_mean_ (C) and ΔTLG+ΔADC_mean_ (D), with cut-off values determined by receiver operating characteristic curve analysis.

Of the 25 patients evaluated, 13 died from lung cancer during the study period, and the median OS was 22.3 months. Kaplan-Meier curves for OS stratified by the cut-off values for CT and ^18^F-FDG PET/MRI findings at 2 weeks and by tumor PD-L1 TPS (<1% vs ≥1%) are shown in figure 4 and online supplemnetary figure S2b. As noted for PFS, the median OS was significantly longer for the patient group with ΔTLG+ΔADC_mean_<16.5 than for the group with ≥16.5 (23.6 vs 4.7 months, p=0.0001, figure 4D), and significant differences in OS were also detected between groups stratified by ΔTLG and ΔADC_mean_ (figure 4B, C). Conversely, no significant differences in OS were detected when patients were stratified by Δdiameter (p=0.077, figure 4A) or PD-L1 TPS (p=0.30, online supplemnetary figure S2b).

Univariate analysis revealed that only ΔTLG+ΔADC_mean_≥16.5 was a significant indicator of shorter OS (HR 9.29, 95% CI 2.49 to 34.62, p=0.00091), whereas ECOG-PS, number of previous regimens, clinical stage, histology and TPS were not significant predictors (table 2).

**Figure 5:**
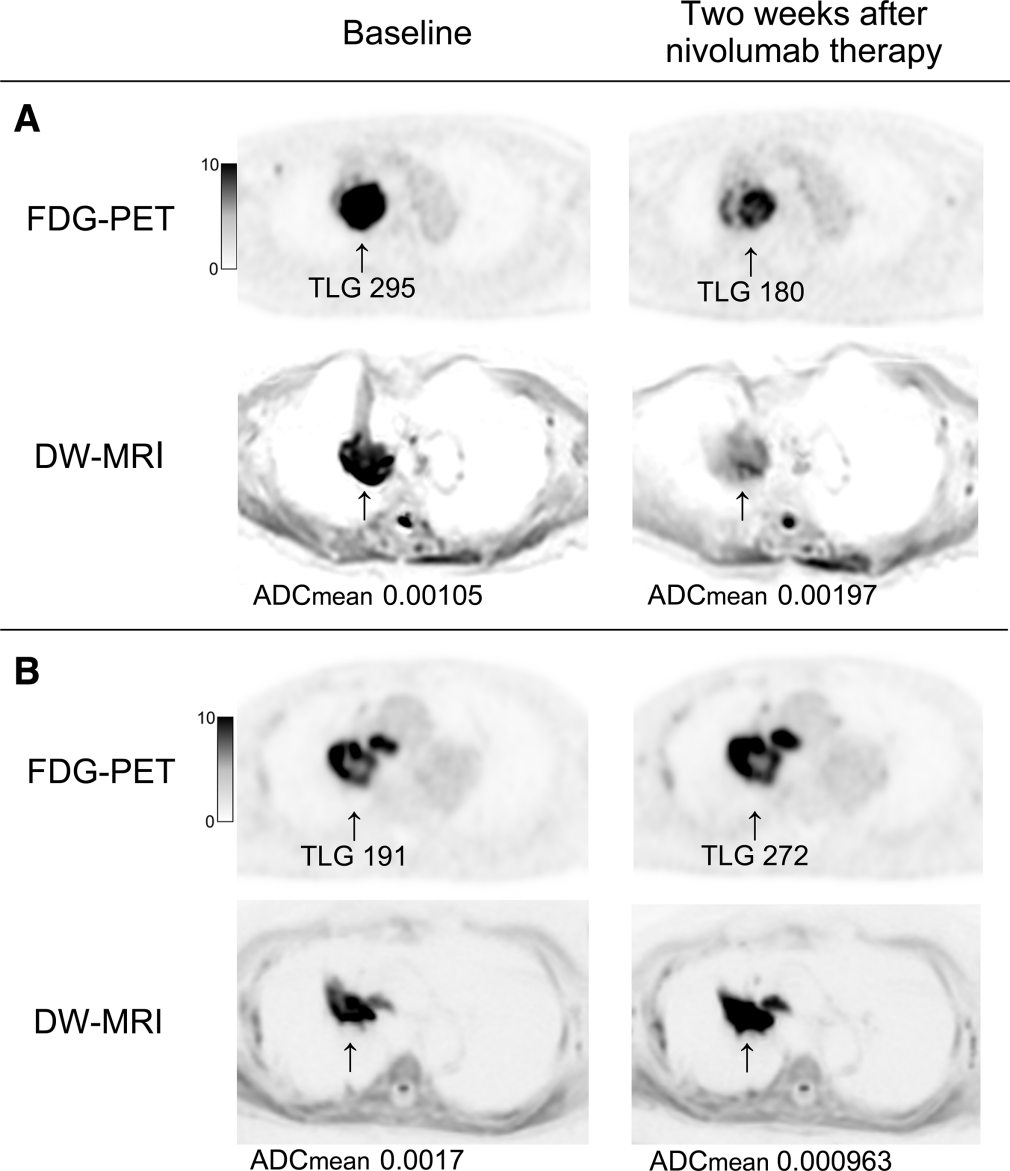
Axial images of [^18^F]-fluoro-2-deoxy-D-glucose positron emission tomography and diffusion-weighted MRI from representative patients. (A) A woman aged 83 years with stage IVB lung adenocarcinoma who achieved a partial response and 9.3 months of progression-free survival after nivolumab therapy. ^18^F-FDG uptake (total lesion glycolysis (TLG)) of the primary lesion in the right upper lobe at baseline decreased by 39% at 2 weeks after nivolumab therapy, while apparent diffusion coefficient (ADC)_mean_ was increased by 46.7%. (B) A man aged 83 years with stage IVA lung adenocarcinoma who experienced progressive disease and short progression-free survival (1.6 months) after nivolumab therapy. TLG and ADC_mean_ were increased by 42.4% and decreased by 43.3%, respectively, after nivolumab therapy.

Figure 5 shows ^18^F-FDG PET/MRI images of two representative patients with PR and PD after nivolumab therapy.

## Discussion

In this prospective study of 25 patients with previously treated NSCLC, we found that changes in TLG, ADC_mean_ and TLG+ADC_mean_ between baseline and 2 weeks of nivolumab treatment were early predictive biomarkers for response, with ΔTLG+ΔADC_mean_ having the best discriminative power. To the best of our knowledge, this is the first report of the predictive utility of integrated ^18^F-FDG PET/MRI findings in this patient population, and additionally, the first report of changes in PET or MRI findings after a single dose of any immune-checkpoint inhibitor in patients with malignant tumors.

In recent years, ^18^F-FDG PET imaging has also been used as a predictive tool for the response to immune-checkpoint inhibitors in patients with melanoma, NSCLC and Hodgkin’s lymphoma.^18–21^ In those studies, the intervals between the baseline (before treatment) and first PET evaluation varied between 3–4 weeks and 1 year. The European Association of Nuclear Medicine symposium recommended that the first response evaluation PET scan should be performed after 8–9 weeks of immunotherapy, which is generally after three or four treatment cycles.^22^ However, two phase III studies of nivolumab for patients with NSCLC showed that the median time to response was only 2.1–2.2 months,^2 3^ which suggests that the recommended time of first response evaluation may be suboptimal for this patient population. Because our findings indicate that changes in integrated ^18^F-FDG PET/MRI parameters can successfully capture the response to nivolumab after a single dose, this method could be used to assist clinicians in designing the optimal therapeutic strategy for an individual patient, thus reducing ineffective treatment and associated drug costs.

Immune-checkpoint inhibitors have emerged as standard therapies for various types of malignant tumors. As noted earlier, a well-recognized atypical manifestation of immune-checkpoint therapy is the so-called tumor pseudoprogression. In patients with NSCLC, the reported rate of pseudoprogression is small (0.6%–4.7%)^23–25^; however, an increase in tumor size that does not meet the RECIST criteria of PD may be seen more frequently. Moreover, a recent study described the morphological and pathological changes after neoadjuvant nivolumab in patients with resectable NSCLC.^26^ In this report, despite apparent tumor enlargement on CT after preoperative administration of nivolumab, some patients exhibited major pathological responses in the resected specimens. Indeed, in our study, 4 of 16 (25%) patients with disease control (CR, PR or SD by RECIST) showed a Δdiameter of ≥10%. This phenomenon can potentially confound CT-based assessment of the early response to immune-checkpoint inhibitors.

One important problem in the use of ^18^F-FDG PET for the diagnosis of malignancy is false positive findings due to inflammatory disease and granulomas.^27–29^ Additionally, in the case of the early response to immune-checkpoint inhibitors, ^18^F-FDG PET can be complicated by the antitumor immune response and enhanced CD8^+^ T-cell infiltration in and around the lesions.^5 26 30^ In the present study, for example, we found that three of the nine responders (PR or CR) exhibited ΔTLG of >20% between baseline and 2 weeks, and three of the nine patients with PD showed a >50% increase in TLG. Therefore, it is important to discriminate between ‘metabolic’ pseudoprogression and authentic disease progression when increased ^18^F-FDG uptake in the lesion is observed after nivolumab treatment.

Diffusion-weighted-MRI is another imaging method used to diagnose and assess various malignant tumors. Water diffusion in tumors can be quantified by ADC, and this has recently been reported to correlate negatively with cell density^12 31 32^ and cell proliferation^33 34^ in several tumor types. Serial changes in ADC have previously been used to analyze the early response of patients with lung cancer to chemotherapy and chemoradiotherapy.^7 35^ However, little is known about how MRI parameters change after treatment with immune-checkpoint inhibitors. In the present study, we found that ADC_mean_ was significantly increased in patients with non-PD compared with PD. The early antitumor response to immune-checkpoint inhibitors is thought to reflect inflammation and edema induced by transient immune cell infiltration,^36^ and non-responders are therefore not expected to show changes in cell density or proliferation in the lesions. Based on these considerations, increased ADC_mean_ (ie, negative ΔADC_mean_) could represent a change in intratumoral cell density and proliferation and may thus be a useful parameter for the assessment of early response to immune-checkpoint inhibitors.

Our study found that ΔTLG+ΔADC_mean_ was the most discriminative ^18^F-FDG PET/MRI parameter for distinguishing between patients with non-PD and PD. Two patients exhibited an increase in ΔTLG between scans of >20% and later achieved a PR (figure 1C), which represented metabolic pseudoprogression. Conversely, these patients showed a decrease in ΔADC_mean_, which resulted in ΔTLG+ΔADC_mean_ below the cut-off value (<16.5, figure 1E). The amelioration of predictive value of ΔTLG+ΔADC_mean_ may result from the decreased ΔADC_mean_, which counteracts the increased ΔTLG in the lesion with metabolic pseudoprogression.

Although PET/CT is recommended as a pretreatment evaluation for staging of patients with NSCLC,^4^ PET/MRI may have some advantages in detecting bone or brain metastases that are frequently seen in patients with NSCLC, as well as accurately combining the metabolic data obtained with the PET image with information about tumor cellularity from MRI. Furthermore, PET/MRI involves reduced exposure to radiation compared with PET/CT. Thus, serial PET/MRI could have widespread utility for evaluating the early response of various malignant tumors to immune-checkpoint inhibitors. However, PET/MRI has some limitations. First, the acquisition time of PET/MRI is longer than PET/CT (30 vs 15 min, in our institution). Second, because the cost of PET/MRI system is higher compared with PET/CT, PET/MRI systems are currently not widespread. Therefore, the predictive utility for the response to immune-checkpoint inhibitors using serial PET/CT and MRI should be verified.

Tumor PD-L1 expression level (measured as TPS) is a recognized predictive biomarker for the efficacy of anti-PD-1 therapy. However, in our study, TPS (<1% vs ≥1%) was not a significant predictor of PFS in nivolumab-treated patients. Some clinical trials of PD-L1 antibodies have reported response rates of only 30%–45%, even in patients with high tumor PD-L1 expression (≥50% TPS).^37 38^ Conversely, approximately 10% of patients with tumors lacking PD-L1 expression respond favorably to nivolumab.^38 39^ These unexpected responses suggest that tumor PD-L1 expression is highly heterogeneous, which renders PD-L1 IHC of biopsy samples an unreliable predictor of response. Indeed, PD-L1 expression has been shown to be heterogeneous within and among lesions^40 41^ and to change after chemotherapy.^42^

In the present study, we found that ΔTLG was a better predictor than ΔSUV_max_ of nivolumab efficacy, which is consistent with some previous studies on the ability of early phase PET to predict the response to immune-checkpoint inhibitor treatment.^20 43^ This finding may reflect heterogeneity in tumor infiltration of inflammatory cells in response to nivolumab treatment, which, in turn, may depend on the heterogeneity of tumor PD-L1 expression. Therefore, SUV_max_ after nivolumab treatment may reflect focal inflammation rather than tumor metabolic activity. Thus, despite being a simple and widespread method, measurement of SUV_max_ or SUL peak might be inappropriate for the early phase assessment of the tumor response to immune-checkpoint inhibitors.^8 44^

Our study has several limitations. The reliability of the cut-off value of this study is not certain because of its preliminary nature and the small sample size; thus, a validation cohort in a larger population is needed to confirm our findings. Furthermore, we could not accurately conclude the correlation between patients’ characteristics, such as age, ECOG-PS and tumor PD-L1 expression, and outcomes under nivolumab from this study. Additionally, we evaluated the response of patients with NSCLC to nivolumab, and the data may not be generalizable to other cancer types or immune-checkpoint inhibitors, such as cytotoxic T-lymphocyte antigen-4 targeted therapies. Further research is thus necessary to confirm our findings and to establish the utility of ^18^F-FDG PET/MRI for early responses to various types of immune-checkpoint inhibitors.

## Conclusion

This prospective study showed that changes in TLG and ADC_mean_ measured by integrated ^18^F-FDG PET/MRI as early as 2 weeks after treatment initiation could have utility as predictors of the response and survival of patients with NSCLC treated with nivolumab. Although further clinical research is needed to confirm our results, our findings suggest that multidimensional functional imaging using integrated PET/MRI holds promise as a method to evaluate the activity of the intratumoral environment and predict the response to treatment with immune-checkpoint inhibitors.
