# Supplementary material for: Predictive value of integrated 18F-FDG PET/MRI in the early response to nivolumab in patients with previously treated non-small cell lung cancer
Source: J Immunother Cancer. 2020 Apr 28;8(1):e000349. doi: 10.1136/jitc-2019-000349 (PMC7213911; doi:10.1136/jitc-2019-000349)
Supplement: Supplementary data [file jitc-2019-000349supp001.pdf]

Additional file 1

Table S1. Changes from baseline in CT and PET/MRI parameters according to confirmed treatment response

	All subjects, N = 25	Non-progressive disease, N = 16	Progressive disease, N = 9	P value[a]
Δdiameter, %	2.2 (-17.7–18.9)	-1.5 (-17.7–18.9)	4.1 (-9.4–16.9)	0.52
ΔSUV_{max}, %	1.6 (-73.8–59.6)	1.7 (-73.8–59.6)	-1.1 (-17.9–48.9)	0.68
ΔSUL peak, %	5.2 (-80.2–63.6)	3.7 (-80.2–34.5)	5.2 (-17.7–63.6)	0.93
ΔTLG, %	10.0 (-80.7–140.2)	-1.0 (-80.7–41.3)	23.1 (-8.5–140.2)	0.008
ΔADC_{mean}, %	-9.1 (-87.6–21.2)	-17.1 (-87.6–8.4)	2.4 (-13.1–21.2)	0.0006
ΔTLG+ΔADC_{mean}	-0.2 (-126.8–142.3)	-15.9 (-126.8–49.7)	30.6 (-2.3–142.3)	0.0001

[a]P values compare the non-progressive and progressive disease groups.

Umeda Y, *et al. J Immunother Cancer* 2020; 8:e000349. doi: 10.1136/jitc-2019-000349
