# Supplementary material for: Predictive value of integrated 18F-FDG PET/MRI in the early response to nivolumab in patients with previously treated non-small cell lung cancer
Source: J Immunother Cancer. 2020 Apr 28;8(1):e000349. doi: 10.1136/jitc-2019-000349 (PMC7213911; doi:10.1136/jitc-2019-000349)
Supplement: Supplementary data [file jitc-2019-000349supp002.pdf]

Additional file 2

Figure S1.

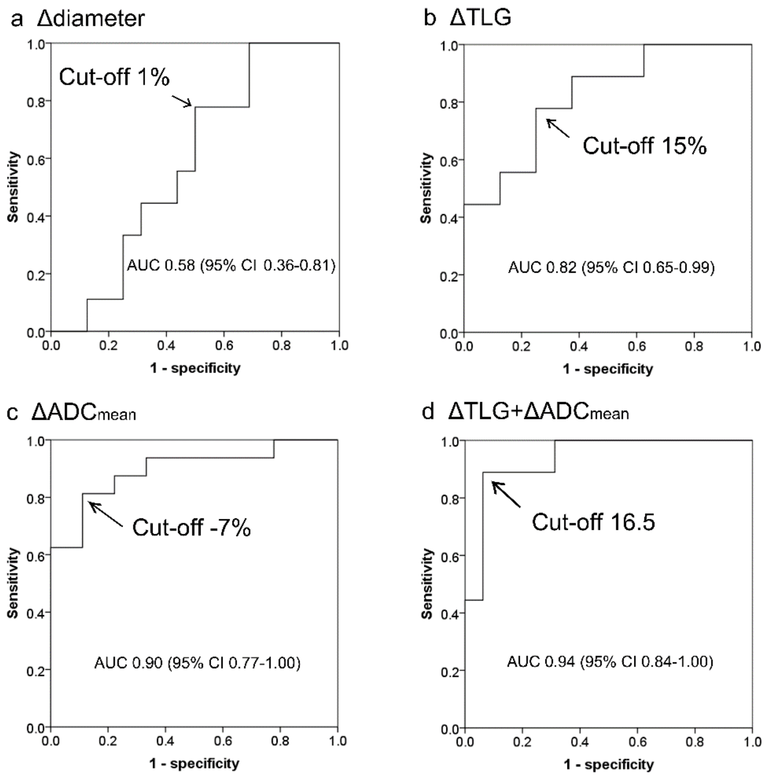

Figure S1. ROC curve analysis of ^{18}F-FDG PET/MRI parameters. Curves were constructed to determine the ability of Δdiameter (a), ΔTLG (b), ΔADC_{mean} (c), and $\Delta TLG+\Delta ADC_{mean}$ (d) to predict disease progression after nivolumab therapy. The optimal cut-off values were estimated to be (a) 1%, (b) 15%, (c) −7%, and (d) 16.5.

Umeda Y, *et al. J Immunother Cancer* 2020; 8:e000349. doi: 10.1136/jitc-2019-000349
