# Supplementary material for: Predictive value of integrated 18F-FDG PET/MRI in the early response to nivolumab in patients with previously treated non-small cell lung cancer
Source: J Immunother Cancer. 2020 Apr 28;8(1):e000349. doi: 10.1136/jitc-2019-000349 (PMC7213911; doi:10.1136/jitc-2019-000349)
Supplement: Supplementary data [file jitc-2019-000349supp003.pdf]

Additional file 3

Table S2 Power of serial CT and PET/MRI parameters to predict progressive disease

	Cut-off (%)	Sensitivity % (N)	Specificity % (N)	PPV % (N)	NPV % (N)	Accuracy % (N)
RECIST 1.1, Δdiameter	20	100 (16/16)	0.0 (0/9)	64.0 (16/25)	0.0 (0/0)	64.0 (16/25)
PERCIST, ΔSUL peak	30	93.8 (15/16)	11.1 (1/9)	65.2 (15/23)	50.0 (1/2)	64.0 (16/25)
EORTC, ΔSUV_{max}	25	81.3 (13/16)	11.1 (1/9)	61.9 (13/21)	25.0 (1/4)	56.0 (14/25)
Δdiameter	1	50.0 (8/16)	77.8 (7/9)	80.0 (8/10)	46.7 (7/15)	60.0 (15/25)
ΔTLG	15	75.0 (12/16)	77.8 (7/9)	85.7 (12/14)	63.6 (7/11)	76.0 (19/25)
ΔADC_{mean}	-7	81.3 (13/16)	88.9 (8/9)	92.9 (13/14)	72.7 (8/11)	84.0 (21/25)
$\Delta TLG+\Delta ADC_{mean}$	16.5	93.8 (15/16)	88.9 (8/9)	93.8 (15/16)	88.9 (8/9)	92.0 (23/25)[†]

RECIST, Response Evaluation Criteria in Solid Tumor; PERCIST, Positron Emission Tomography Response Criteria in Solid Tumors; EORTC, European Organisation for the Research and Treatment of Cancer; TLG, total lesion glycolysis; ADC_{mean}, average apparent diffusion coefficient; PPV, positive predictive value; NPV, negative predictive value

[†]$p = 0.027$ compared with Δdiameter by McNemar’s test.

Umeda Y, *et al. J Immunother Cancer* 2020; 8:e000349. doi: 10.1136/jitc-2019-000349
