# Supplementary material for: Predictive value of integrated 18F-FDG PET/MRI in the early response to nivolumab in patients with previously treated non-small cell lung cancer
Source: J Immunother Cancer. 2020 Apr 28;8(1):e000349. doi: 10.1136/jitc-2019-000349 (PMC7213911; doi:10.1136/jitc-2019-000349)
Supplement: Supplementary data [file jitc-2019-000349supp004.pdf]

Additional file 4

Figure S2.

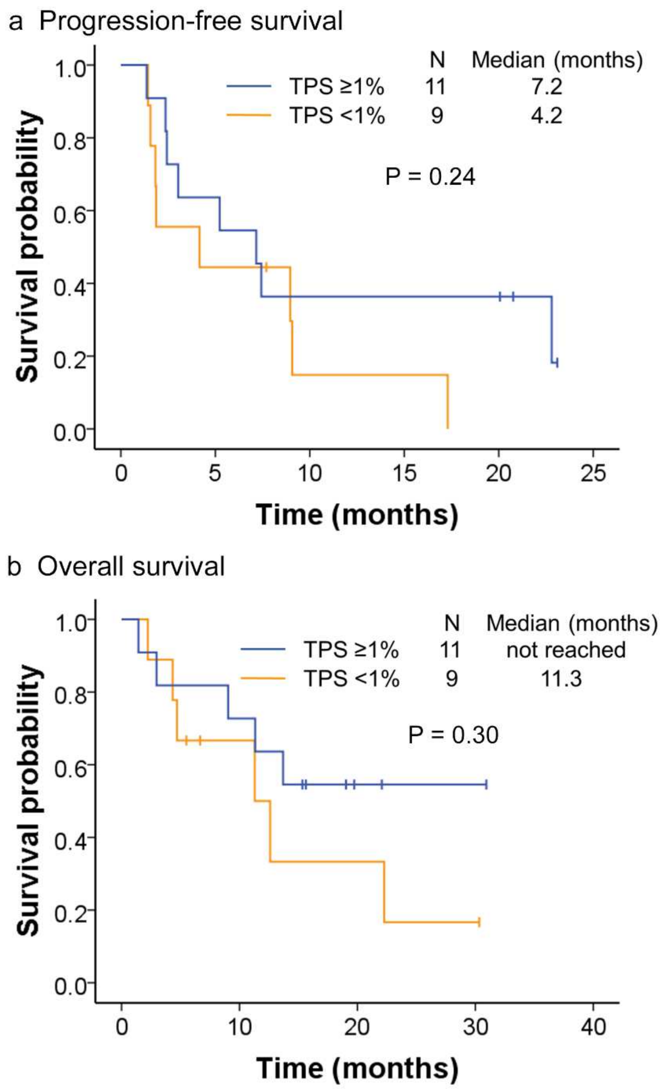

Figure S2. Kaplan–Meier curves of progression-free survival (a) and overall survival (b) of nivolumab-treated patients according to tumor PD-L1 expression.

Umeda Y, *et al. J Immunother Cancer* 2020; 8:e000349. doi: 10.1136/jitc-2019-000349
